# Field application of an improved protocol for environmental DNA extraction, purification, and measurement using Sterivex filter

**DOI:** 10.1038/s41598-020-77304-7

**Published:** 2020-12-09

**Authors:** Marty Kwok-Shing Wong, Mako Nakao, Susumu Hyodo

**Affiliations:** 1 Laboratory of Physiology, Atmosphere and Ocean Research Institute, The University of Tokyo, 5-1-5 Kashiwanoha, Kashiwa City, Chiba 277-8564 Japan; 2 Laboratory of Marine Ecosystem Change Analysis, Field Science Center for Northern Biosphere, Hokkaido University, Hakodate Research Center for Fisheries and Oceans, 20-5 Benten-Cho, Hakodate City, Hokkaido 040-0051 Japan

**Keywords:** Ecosystem ecology, Molecular ecology, Isolation, separation and purification, Biological techniques, Ecology, Molecular biology, Ecology, Environmental sciences, Ocean sciences

## Abstract

Environmental DNA (eDNA) is increasingly popular as a useful non-invasive method to monitor and study biodiversity and community structure in freshwater and marine environments. To effectively extract eDNA from the filter surface is a fundamental factor determining the representativeness of the samples. We improved the eDNA extraction efficiency of an established Sterivex method by 12- to 16-fold using a larger volume of lysis buffer mix coupled with backflushing the cartridges. The DNeasy extraction column could be overloaded when the environmental sample input is high, possibly due to a higher nonspecific binding present in environmental samples, thus resulting in a relatively lower quantity measured. Therefore, we included an internal control DNA in the extraction to monitor the extraction and purification efficiencies in field samples, which is crucial for quantification of original eDNA concentration. The use of Takara Probe qPCR Mix supplemented with protein-based additives improved the robustness of the real time PCR assay on inhibitor-rich environmental samples, but prior purification by Qiagen PowerClean Pro Cleanup kit could be essential for inhibitor-rich water samples, even though the recovery rate was unexpectedly low (average 33.0%). The improved extraction and quantification complement the qualitative analyses including metabarcoding and metagenomics in field application.

## Introduction

The use of environmental DNA (eDNA) to monitor the biodiversity of the environment, community structure of a population, and exotic species invasion, has been popular in recent years^1–3^. To collect eDNA from water samples, various filtration systems were established including glass microfiber filters (GF/F), cellulose nitrate (CN) filters, and Sterivex cartridges^4^. Our group previously adopted a GF/F system to investigate the eDNA distribution and downstream migration pattern of juvenile chum salmon in Otsuchi Bay of Iwate Prefecture of Japan^5^, which involved large numbers of eDNA extractions (over 800 samples in year 2018). We also demonstrated a high correlation between chum salmon density and eDNA concentration in field and laboratory water samples using qPCR quantification. For future eDNA studies and automation system development, an enclosed filter system such as the Sterivex cartridge is preferable^6^. These cartridges are generally more expensive, but can reduce contamination from handling and are easy to use with syringe attachment or with vacuum aspiration device. Surveys on fish diversity by metabarcoding and specific assays were performed in water systems using the eDNA samples collected by Sterivex cartridges^7–12^.

Before performing large scale eDNA surveys using Sterivex cartridges, the optimization of extraction method is essential. We investigated an eDNA extraction method for Sterivex cartridges^13^ that was widely used in other eDNA studies^2,8,9,14–17^. Most eDNA studies with metabarcoding or metagenomic methods focused mainly on qualitative analysis (e.g., relative abundance of a certain family in the water sample), while the absolute quantities are often not considered. However, to study the dynamics of organisms in the environment, one must consider both qualitative and quantitative aspects of the eDNA samples. The extraction of eDNA from the cartridges is the fundamental step determining the value of the sample, and is therefore deserving of investment in validation. Furthermore, we experienced high levels of environmental inhibitors that affect the PCR reaction, thereby necessitating additional sample purification steps^5^. The extraction efficiencies of eDNA could be variable due to unpredictable loading on the filter and inhibitory substances present in the water sample. Therefore, high uniform eDNA extraction are essential for reliably estimating the original eDNA quantities in the water, allowing the quantitative inference of the organisms in the environments. To verify whether the commonly used protocol is suitable for quantitative analysis, we set out to investigate the efficiency of eDNA extraction and purification using indoor aquarium water holding chum salmon, and then apply the protocol to field samples for validation and subsequent improvement. We modified the protocol to maximize the extraction efficiency with minimal steps while maintaining the extraction at a reasonable cost.

## Results

### Effects of lysis buffer volume and method of introduction

**Figure 1:**
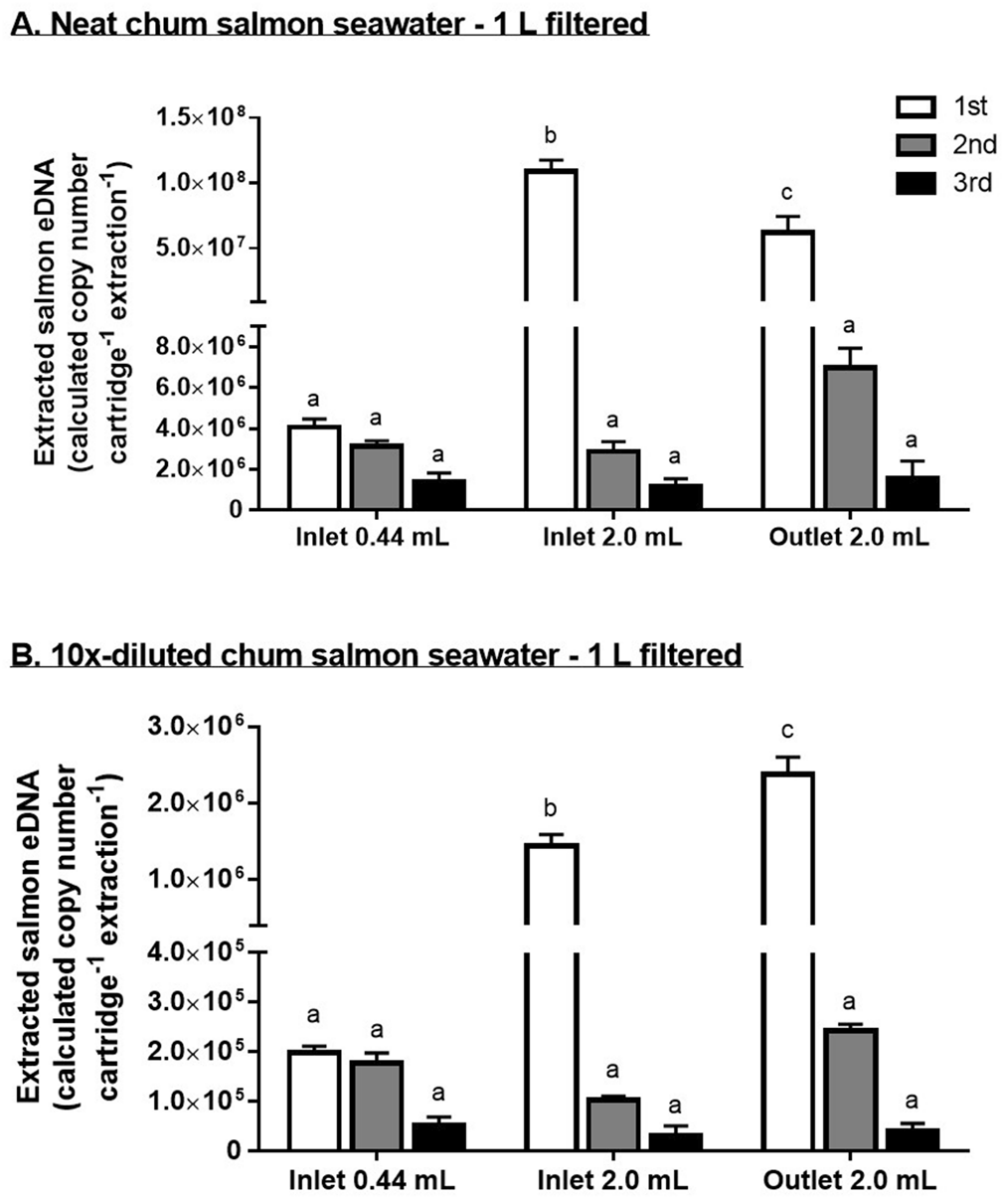
Workflow and various protocols examined. Two concentrations of chum salmon tank water (neat vs. 10×-diluted) were filtered to simulate low and high sample inputs. Three protocols are presented schematically to indicate the modifications (refer to main text for details). A spin column fitted to a 50 mL centrifuge tube is used to collect the lysate from the Sterivex cartridge. Ethanol is added to the lysate and the mixture is loaded on the DNeasy column.

We extracted each cartridge three times to examine whether the protocols are effective to recover most of the eDNA attached on the column in a single extraction (Fig. 1). The original protocol (Protocol 1) used a relatively small volume of lysis buffer (0.44 mL) and utilized a rotation system to maximize the contact of the buffer to the filtration surface. We tested three consecutive extractions and found that similar amounts of salmon eDNA could be collected in the first and second extractions; collection of salmon eDNA in the third extraction was approximately one-third the amount recovered in the first extraction (Fig. 1A). In Protocol 2, we tested a larger volume of lysis buffer (2.0 mL), which is enough to cover the entire filtration surface of the cartridge. The first extraction resulted in a 27-fold increase in salmon eDNA extraction (Fig. 1A) when compared to those of Protocol 1. In the second and third extraction, similar amounts were obtained as those of Protocol 1. In Protocol 3, we tested whether backwash from the outlet with the larger volume of lysis buffer (2.0 mL) can further enhance the total salmon eDNA recovery. The first extraction resulted in a 16-fold increase in eDNA compared to that of Protocol 1, but the quantity was significantly lower (43% less) than that of Protocol 2. The yield of second extraction in Protocol 3 was double to those of protocols 1 and 2, while the yields of third extraction among the three protocols were similar. However, no statistical significance was found among the second and third extraction among all 3 protocols. From the first extraction, the average yield of DNA among the high input groups were 189.3 ± 112.0 ng, 1183.3 ± 112.0 ng, and 1183.3 ± 86.6 ng (n = 6 in each case) for Protocol 1, 2, and 3 respectively.

For the low input groups, the salmon water used was 10% of the high input groups. Compared to the salmon eDNA extracted by protocols 1, 2, and 3 in the high input groups, the yields of among low input groups were 4.8%, 1.3%, and 3.8% respectively. The first and second extraction by Protocol 1 resulted in similar quantities, while one-fourth was yielded in the third extraction (Fig. 1B). In Protocol 2, the first extraction resulted in a sevenfold increase in salmon eDNA content, while the eDNA contents of the second and third extraction were approximately one-half to those of Protocol 1. In Protocol 3, the first extraction resulted in a 12-fold increase in salmon eDNA content compared to that of Protocol 1, which was significantly higher than that of Protocol 2. The salmon eDNA contents obtained from second and third extraction were similar to those of Protocol 1. No statistical significance was found among the second and third extraction among all 3 protocols (Fig. 1B). From the first extraction, the average yield of DNA among the low input groups were 392.0 ± 33.2 ng, 401.3 ± 37.8 ng, and 682.0 ± 35.5 ng (n = 6 in each case) for Protocol 1, 2, and 3 respectively.

### Effects of input quantity on eDNA extraction

**Figure 2:**
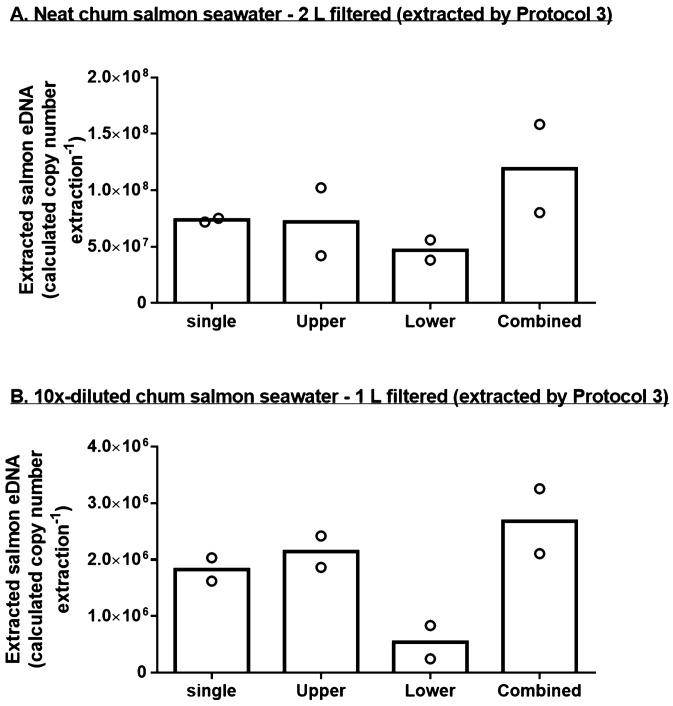
Calculated eDNA concentrations extracted by various protocols at (**A**) high (neat chum salmon seawater) and (**B**) low (10×-diluted chum salmon seawater) inputs (N = 6). Each cartridge was extracted three times with the same protocol and the eDNA concentration was quantified separately. Data is presented as mean ± S.E.M. For protocol-dependent effects, Tukey’s test was performed after two-way ANOVA and different alphabet letters denote significant difference (*p* < 0.05) among protocols and repeated extraction.

As we observed a lower extraction efficiency of chum salmon eDNA in the backwash group when the input was high, we suspected that the DNeasy column may have been saturated by the samples, leading to a proportional loss in the flowthrough during the binding step. To test this, we connected two DNeasy columns in series to test how much eDNA was lost from the first column in high and low inputs. To ensure the input was sufficiently high, we filtered 2 L of neat salmon seawater instead of 1 L used in the protocol developing experiments. The filtered cartridges were extracted by Protocol 3 described above. In case of high sample inputs, the upper and lower columns adsorbed similar amount of salmon eDNA as in single column (Fig. 2A), suggesting that capacity of DNeasy column was saturated by the high inputs. For low inputs, the upper DNeasy column sufficiently adsorbed the salmon eDNA from the extracted buffer, resulting in a low concentration of salmon eDNA detected in the lower column (Fig. 2B).

As we anticipate the possibility of saturated adsorption by the DNeasy column in environmental samples, we estimated the extraction efficiency by introducing a fixed quantity of internal control DNA in each extraction in the field samples. The detection rates of the internal control DNA quantified by qPCR were 11.2–62.8%, with an average of 35.9 ± 2.5% among the 34 random environmental samples.

### Efficiency of PowerClean Pro Cleanup kit

**Figure 3:**
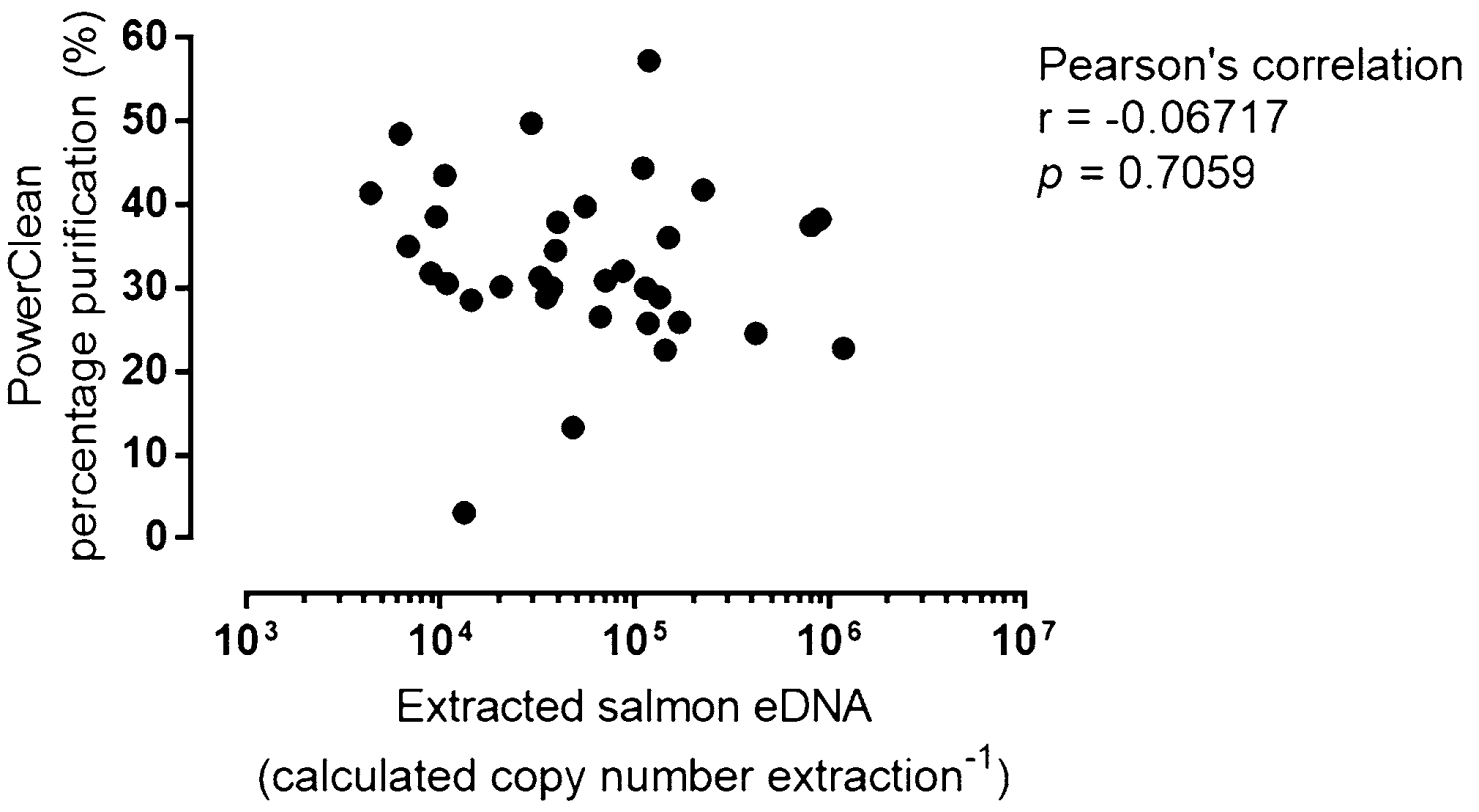
Calculated Chum salmon eDNA concentrations extracted by single or double DNeasy columns at (**A**) high (neat chum salmon seawater—2 L) and (**B**) low (10×-diluted chum salmon seawater—1 L) eDNA inputs (N = 2). Sterivex cartridges were extracted by Protocol 3 described in the text. Each column was eluted and chum salmon eDNA was quantified separately. Data is presented as mean with sample values.

To estimate the purification efficiency of PowerClean kit, we compared the measured concentration of salmon eDNA in the random field samples before and after the purification. The measured concentration of the extracted salmon eDNA among the field samples ranged from 4.38 × 10^3^–1.18 × 10^6^ copies L^−1^. After PowerClean purification, the measured concentration ranged from 4.11 × 10^2^–3.42 × 10^5^ copies L^−1^. The measured salmon eDNA was average 33.0% after the PowerClean purification. The Pearson’s correlation (r = −0.067; *p* = 0.7059) did not indicate any correlation between the purification efficiencies and initial salmon eDNA concentrations from field water samples (Fig. 3). Internal control was included in the lysis buffer for the extraction of the field samples, thus allowing the estimation of extraction and purification efficiencies. Among the 34 random environmental samples, the percentage recovery of the internal control DNA ranged from 1.1% to 40.4%, with an average of 19.4 ± 1.4% after extraction and purification processes.

### PCR optimization without PowerClean purification

**Figure 4:**
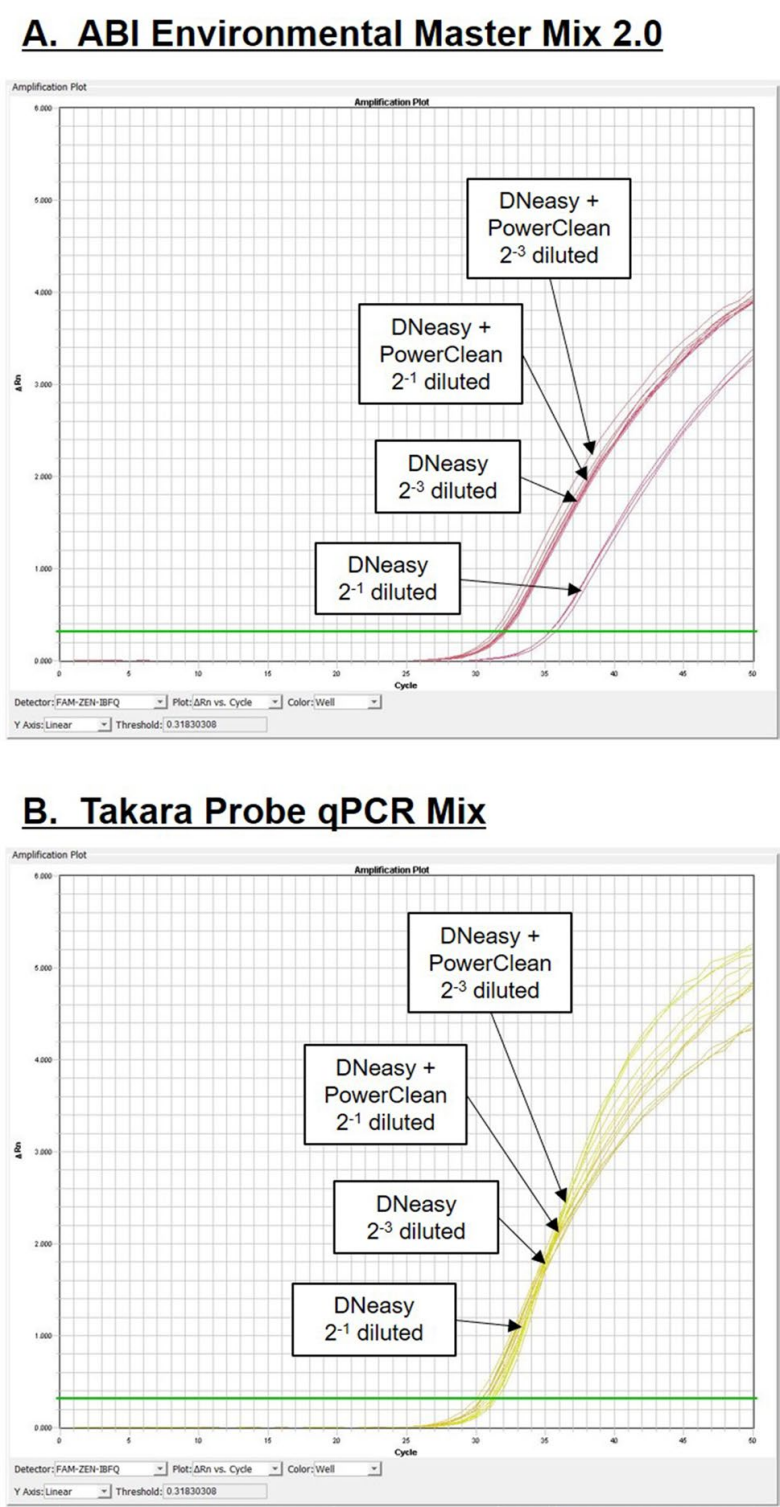
Correlation between PowerClean percentage purification and input calculated salmon eDNA concentration in random field water samples. No significant Pearson’s correlation was found between input eDNA concentrations and percentage purification by PowerClean kit.

As we observed relatively low and variable purification rate of PowerClean purification, we explored the options whether the PCR reaction can be optimized using the Takara Probe qPCR Mix alone since the reagent has a higher tolerance for environmental contaminants. The PCR inhibitor tolerance was compared between ABI Environmental Master Mix and Takara Probe qPCR Mix using a field sample from Murohama, which was shown to contain substances strong enough to inhibit the reaction with Environmental Master Mix^5^. Serial dilution of the sample was spiked with salmon eDNA, and the amplification curve shifted to the right in case of 2^−1^ dilution, indicating that the PCR amplification was inhibited. The reaction could be freed from inhibition when the field sample was diluted to 2^−3^ or when PowerClean purification was performed (Fig. 4A). Using the same samples and setup, we showed that the Takara Probe qPCR Mix has a higher robustness as the PCR reaction was not inhibited at 2^−1^ dilution even without PowerClean purification (Fig. 4B).

**Figure 5:**
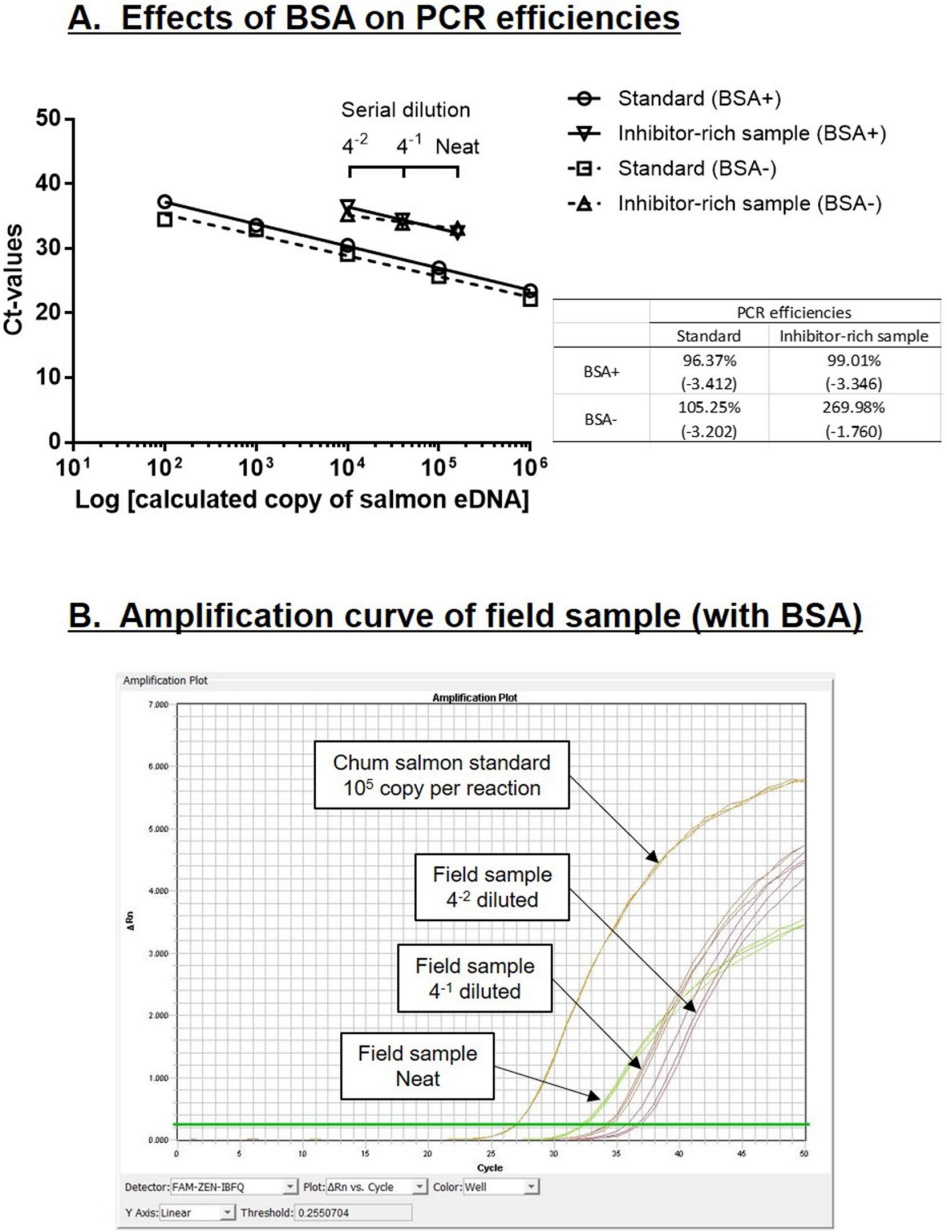
Comparison of PCR robustness between (**A**) ABI Environmental Master Mix 2.0 and (**B**) Takara Probe qPCR Mix on PCR inhibitor tolerance. The real-time PCR was performed with a field sample known to contain high level of PCR inhibitors, and each reaction was spiked with a fixed amount of chum salmon eDNA to monitor the amplification profile. The field sample was serially diluted to reduce the inhibitor influence. PCR efficiency is considered to be compromised when the amplification curve shifts to right or when no amplification is observed.

Using the 34 random environmental samples, we tested the performance of Takara Probe qPCR Mix on the measuring of field samples. Relative chum salmon eDNA in the field samples could be measured directly using the Takara Probe qPCR Mix, but PCR inhibitors in the field samples could lower the accuracy of the analysis. In order to test for PCR inhibition, we spiked 10^5^ copies of standard chum salmon DNA in each PCR reaction and examined the quantities obtained from normal and spiked reactions. PCR inhibition was evident when a lower value of chum salmon standard DNA was measured. The percentage detection of the spiked salmon DNA ranged from 37.0% to 133.4%, with an average of 103.6 ± 3.5%. The sample with the lowest percentage detection (37.0%) was inhibitor-rich, and we examined how the environmental contaminants could have affected the PCR reaction by comparing the PCR efficiencies between sample and standard. PCR efficiency over 100% is often caused by the presence of inhibitors in samples^18^ as a higher Ct value is obtained for the concentrated samples in the dilution series, where contaminants in the samples are more concentrated. This leads to a flatter slope in the dilution curve and thus a higher efficiency is calculated. We showed that the calculated PCR efficiency of the inhibitor-rich sample was high (269.98%), suggesting that the PCR reaction was clearly inhibited by the environmental contaminants (Fig. 5A).

For the BSA-containing protocol, serially diluted inhibitor-rich sample was used for measurement of salmon eDNA to calculate the PCR efficiency. The slope of the dilution curve of the inhibitor-rich sample shifted from − 1.760 to − 3.346, which was similar to that of the standard (-3.412), indicating that the addition of BSA improved PCR efficiency (99.01%) among contaminated samples (Fig. 5A). Although the PCR efficiency was highly improved by the BSA addition, the amplification plot of the inhibitor-rich sample at neat concentration suggested that the PCR condition was not optimal as the amplification curve shifted downward (Fig. 5B), indicating that slight PCR inhibition remained. The amplification curves of diluted samples are parallel to that of standard (Fig. 5B), indicating that the PCR inhibition was marginal and thus PCR efficiency was not heavily affected. In addition to BSA, we tested other chemical and protein-based additives including DMSO, Tween 20, formamide, and GP32 protein (Table 1). Most chemical additives did not rescue the PCR inhibition as the saturated fluorescence and the slopes of PCR amplification plots were similar to that of no additive. Addition of GP32 protein partially rescued the PCR inhibition with an improved saturated fluorescence and slope closer to that of standard.

**Table 1:** Effects of various additives on the real time PCR amplification of a pooled environmental sample rich in inhibitors.

Additives for qPCR	Saturated fluroscence intensity (AU)			Slope of PCR amplification curve		
	Chum salmon standard (10^5^ copy per reaction)	Inhibitor-rich sample	% decrease	Chum salmon standard (10^5^ copy per reaction)	Inhibitor-rich sample	% decrease
No additive	12,860	8954	30.4	6.92	5.08	26.7
BSA (1 mg/mL)	12,726	9207	27.7	6.74	5.23	22.4
DMSO (2%)	11,946	8872	25.7	6.31	4.91	22.1
Tween 20 (0.5%)	12,619	9614	23.8	7.08	5.56	21.4
Formamide (0.01%)	11,815	8549	27.6	7.23	5.46	24.5
GP-32 (0.1 mg/mL)	11,540	9932	13.9	7.14	6.60	7.6

Slope of the PCR amplification curve is calculated by linear regression of the steepest portion.

## Discussion

We investigated an established eDNA extraction method on Sterivex cartridge and modified the protocol to optimize the eDNA extraction efficiency and to reduce intensity of labor. The use of metabarcoding and metagenome to analyze the population complexity in eDNA is increasingly popular and can show the relationship between environmental properties and biodiversity^1,7,19^. Depends on assays, the qualitative method may have limited resolution to species diversity and are sometimes sensitive up to family level^20,21^. On the other hand, quantitative methods using qPCR on certain species not only can detect the presence of the target species, but also infers the relative abundance of the species in the environment, which complements the limitation of metabarcoding analysis^5,22^. In open oceans, the eDNA concentrations are often low, and filtering large volumes of water is required to accumulate sufficient amounts of eDNA. For accurate representative value of the eDNA sample, the eDNA extraction from the filtration system should be effective, reliable, and testable.

Our group studied the chum salmon eDNA changes in Otsuchi Bay of Iwate Prefecture using GF/F filter in 2017–2018^5^. To pave a way for future automation in eDNA collection and extraction, we adopted Sterivex cartridges to collect the eDNA samples from environmental water. We first tested an established eDNA extraction method^13^, but found some high variations in extracted eDNA quantity relative to input quantity from preliminary experiments using indoor aquarium water (unpublished observations). While the quantitative analysis is important for estimating the scale of salmon migration, we therefore set out in this study to optimize eDNA extraction efficiency and improve workflow.

**Figure 6:**
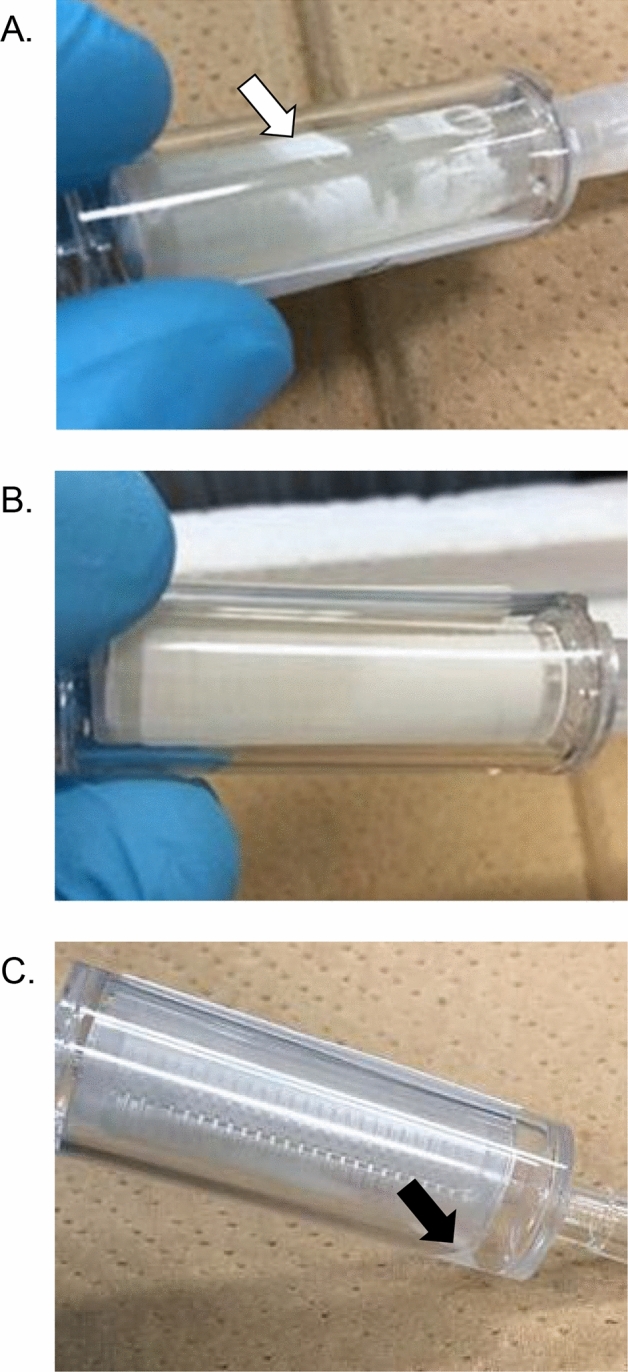
Effects of BSA on the PCR efficiencies of Takara Probe qPCR Mix. (**A**) A field sample known to contain high-level PCR inhibitors was serially diluted and measured by Takara Probe qPCR Mix with or without BSA supplementation. The upper scale bar represents dilution of the field samples. The embedded table shows the PCR efficiency of each dilution curve, and the slope is given in parentheses. PCR efficiency higher than 100% (or slope higher than − 3.322) suggests the presence of PCR inhibitors. BSA supplementation increases adsorption of non-specific inhibitors and restores the PCR efficiency in the inhibitor-rich environmental sample. (**B**) PCR amplification curves of an inhibitor-rich environmental sample. The amplification curves of neat sample shifted downwards, suggesting the reaction was compromised but not entirely inhibited. Dilution of environmental samples restored the parallelism of amplification curves to that of standard, suggesting the inhibition was marginal in this specific case.

Increasing the volume of lysis buffer mix (0.44–2.0 mL) dramatically increased the extracted eDNA quantity, suggesting the original protocol was not suitable for quantitative analysis as the eDNA content was largely underestimated and variable. This could be due to the insufficient contact of the lysis buffer mix with the filtering surface, which was evident from the watermark left behind after extraction, suggesting that some filter region was dried (Fig. 6A, B). Repeated extraction did not increase the efficiency, indicating the filter surface has insufficient contact with the lysis buffer during rotating incubation even in repeated extraction. Furthermore, we expected that large particles in the water could be trapped in the pores of the Sterivex cartridge, resulting in poor contact with the lysis buffer mix during incubation. Backwash from the outlet further increased the eDNA extracted from the cartridge (Fig. 1B), demonstrating that the trapped particles were flushed out successfully by the reverse flow. However, we observed that backwash did not further increase the eDNA from the cartridge with high sample input (Fig. 1A), leading us to hypothesize that some other factors could be limiting the extraction or retention of eDNA in high sample inputs.

We demonstrated that the saturation of DNA binding of the DNeasy column may explain the reduced salmon eDNA quantified from the high input group with Protocol 3. When the initial input was high, two consecutive DNeasy columns collected similar amount of salmon eDNA, indicating that large portions of eDNA were lost in the flowthrough of the upper columns, which were caught by the bottom columns (Fig. 2A). The salmon eDNA content in each of the consecutive columns was similar to those of single column, suggesting that the input sample exceeded the maximum capacity of the DNeasy column. When the input is low, however, over 90% of the eDNA could be adsorbed by the upper column. We measured the extracted DNA quantities and found that the values are lower (0.4–1.2 μg) than the stated capacity of the DNeasy column, which has tens of μg DNA adsorption capacity when used in tissue extraction. The results suggested that unknown environmental compounds and contaminants in the environmental samples could contribute to non-specific binding to the silica surface, thus compete with or inhibit the desired DNA binding. Although the DNeasy column has tens of microgram DNA binding capacity, only a fraction of eDNA was bound when the nonspecific binding was high. Our results suggest that DNA concentration may not be sufficient to infer the richness of target eDNA from a sample.

In the field samples, the saturation of DNeasy column is also possible because the water can be rich in plankton, algae, fecal contents, decaying debris, etc.^23,24^, which generate large loadings of eDNA and non-specific binding. However, it is unpredictable whether the extracted sample could have exceeded the capacity of the DNeasy column in field samples. Therefore, monitoring the efficiency of each eDNA extraction using an internal control is necessary to extrapolate the quantity of the original eDNA content from the filtered sample.

In order to overcome the potential saturation of DNeasy column, we tested the use of an internal control (e.g. a fixed quantity of plasmid DNA) introduced to the lysis buffer mix. The percentage recovery of the internal control from extraction to purification allow the calculation of the extraction efficiency in each sample. We tested the internal control in the random field samples and found variable extraction efficiencies (11.2–62.8%) and purification efficiencies (1.1–40.4%) among samples. When calculating the original eDNA concentration in filtered sample, the eDNA copy number of a species should be corrected by the percentage recovery to account for the loss during DNeasy extraction and/or PowerClean purification.

We validated the purification efficiency of PowerClean in this study with the field samples and found no positive correlation among input salmon eDNA and the detection rates after purification (Fig. 3), suggesting that the PowerClean purification was not biased by the input quantities. However, it was surprising that the detection rate was relatively low (average 33.0%). This demonstrated that the addition of internal control is essential to determine the purification efficiency. Variable and low detection rates can greatly impact the quantification of target eDNA in population density estimation^4^, so we re-considered whether purification by PowerClean Kit is avoidable.

Our group previously demonstrated the importance of purification (PowerClean Pro Cleanup kit) on the detection of chum salmon eDNA from the field samples as they carried enormous amount of PCR inhibitors that compromise the qPCR reaction^5,25^. However, among new formulations of PCR reagents that are becoming available on the markets, some are designed to have high tolerance to PCR inhibitors in environmental samples^26^. In our previous study^5^, the ABI Environmental Master Mix 2.0 was robust to perform qPCR on environmental samples purified by PowerClean kit. To avoid the use of PowerClean kit, we tested a new PCR reagent, Takara Probe qPCR Mix, and found that it is more robust to PCR inhibitors present in our samples than ABI Environmental Master Mix 2.0 (Fig. 4). Various types of PCR reagents have different degree of tolerance against known PCR inhibitors including humic, fulvic, and tannic acids^26^ and the use of Takara Probe qPCR Mix could be advantageous specifically to our sampling sites. We explored the possibility of whether the eDNA can be quantified directly after DNeasy extraction without additional purification by the PowerClean kit. The spiked salmon DNA standard in the environmental samples can be measured by qPCR at reasonably high accuracy (103.6 ± 3.5% of spiked DNA) among 34 random environmental samples. Although some samples have relatively low measured values (e.g. 37.0% of spiked DNA), we did not experience “zero measurement”, which was common among the PCR reagents tested previously. Nevertheless, the calculated PCR efficiency of the inhibitor-rich sample (269.98%) was much higher than 100%, which is a common sign of PCR inhibitor presence in the sample^18^. From our experience, addition of BSA in PCR reaction may release a certain level of inhibition as the protein adsorbs the inhibitors, and thus less is available to inhibit the enzymatic reaction. Indeed, when BSA was introduced to the Takara Probe qPCR Mix, the PCR efficiency of the inhibitor-rich sample was restored (Fig. 5A). However, detailed examination of the amplification curves showed that the PCR reaction was hampered in the sample at neat concentration (Fig. 5B), indicating that the inhibition was still affecting the quantification, even after improvement by BSA addition. Dilution of the environmental sample restored the parallelism of amplification curves to that of standard, suggesting the inhibition was marginal in this specific case. In addition to BSA, we further tested chemical and protein additives on the real time PCR reaction containing inhibitor-rich samples. For the nature of the PCR inhibitor from our sampling site, chemical additives did not improve the PCR reaction further. However, addition of GP-32 protein further recused the PCR reaction and it lessened the decrease in saturated fluorescence among the tested additives and generated a PCR amplification curve with a slope similar to that of standard (Table 1). This preliminary result prompts to further tests of PCR additives for tackling inhibitor-rich environmental samples in the future.

At present, the site-specific PCR inhibitor issue was not completely solved and PowerClean purification offers an option to remove the inhibitors despite of its variable and low purification efficiency. We anticipated that water samples from the coastal area are highly contaminated and should be treated by PowerClean purification before qPCR. However, water samples away from shores may contain less PCR inhibitors, and thus PowerClean purification can be removed from the standard procedure to reduce cost and loss in eDNA contents. Furthermore, we strive to develop a robust PCR assay that allow the direct measurement of eDNA without purification. To account for the potential loss of eDNA from extraction and/or purification, normalization against internal control is crucial for estimating the original eDNA contents in the filtered samples.

Besides testing the efficiencies, we also improved the extraction workflow by introducing a spin column that fits into a 50 mL centrifuge tube for collecting the lysate from the Sterivex cartridge. The use of the spin column along with a swing-type rotor allows a higher recovery of lysate, as some lysate often remains in the Sterivex cartridge when centrifuged with an angled rotor (Fig. 6C). A larger volume of lysate can be accommodated by the 50 mL centrifuge tube, which allows a greater choice of the volume of initial digestion. When using parafilm to plug the inlet or outlet, liquid often leaks during incubation and handling, which causes cross contamination and loss of samples. The introduction of stoppers and silicon tube connections improved the extraction procedures and decreased the contamination chances. These improvements together increase the throughput and decrease the labor force for extraction.

In conclusion, we optimized a new protocol for the extraction of eDNA from Sterivex cartridge and for quantification of specific species using real time PCR, focusing on the quantitative aspects. An internal DNA control added at the beginning of the extraction allows the calculation of extraction and purification efficiencies. Larger volumes of lysis buffer mix coupled with backflush increases significantly the extraction efficiency of Sterivex cartridge. A robust PCR mix supplemented with protein-based additives improved the qPCR reaction for inhibitor-rich environmental samples, but purification by PowerClean Kit prior to quantification may be still essential for some inhibitor-rich samples from coastal area. The qPCR assay condition could be specific to our sampling sites and further studies are required to investigate the practicality in other areas where different types of inhibitors exist. This protocol improvises monitoring tools in various levels of the workflow, and thus increases the reliability of the eDNA data to infer the dynamics of environments in quantitative and qualitative perspectives. A boost in extraction yield by 12- to 16-folds could deepen the population or community coverage significantly, allowing the detection of rare organisms which could be easily missed due to low extraction yield.

## Methods

### Fish culture

Fertilized eggs of chum salmon [*Oncorhynchus keta* (Walbaum, 1792)] were obtained from Unosumai hatchery, Iwate, Japan. The eggs were fertilized artificially on 4th December 2018 and eye-stage embryos were transferred to the Atmosphere and Ocean Research Institute, Chiba, Japan. Hatched salmon fries were fed with commercial diet after emergence in freshwater. Natural seawater (SW, salinity 35 ‰) was obtained from the Kuroshio Current at Hachijō-jima. The SW was stored in underground facilities and salmon eDNA was not detectable from the SW stock from in-house experiment. Salmon juveniles were transferred to seawater tanks after 2-months culture in freshwater. The experimental tank contained 500 L seawater with recirculation and temperature control at 14 °C. Twenty-five individuals (fork-length: ca. 20 cm; weight: ca. 50 g) were kept in the tank and the water served as a source of eDNA in the present study. All animal studies were performed according to the Guideline for Care and Use of Animals approved by the Animal Experiment Committee of The University of Tokyo.

### Filtration of water

**Figure 7:**
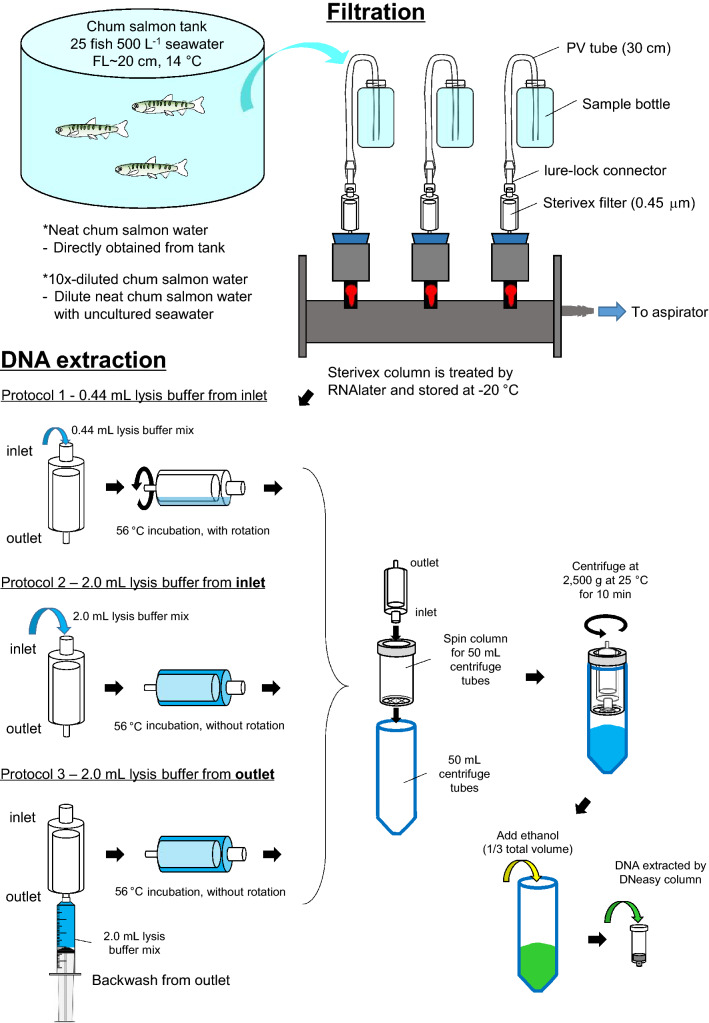
Representative photos of Sterivex cartridges with different extraction protocols. (**A**) A cartridge extracted by 0.44 mL lysis mix protocol. Note the water mark that is present on the filter surface (white arrow), suggesting insufficient contact with the lysis buffer mix. (**B**) A cartridge extracted by 2.0 mL lysis buffer mix with backwash. Note that no water mark is found as in (**A**). (**C**) A cartridge centrifuged by angled rotor. Note that remaining lysate is present (black arrow).

Two concentrations of salmon water were prepared. “Neat salmon water” was obtained directly from the stock tank. “Diluted salmon water” was prepared by diluting the tank water 10 times with seawater that had not been used for any previous fish culture. To standardize filtration conditions, 1 L of salmon water at either concentration was filtered through a Sterivex cartridge (0.45 μm, Millipore SVHV010RS, Merck, Tokyo, Japan) using a vacuum manifold (Manifold KMP-3, Advantec, Tokyo, Japan) attached to an aspirator (Eyela A-1000S, Tokyo Rikakikai, Tokyo, Japan) (Fig. 7). After filtration, the outlet of the Sterivex cartridge was sealed by Parafilm and 1.6 mL of RNAlater was introduced into the cartridge through the inlet. The inlet was subsequently sealed by Parafilm and the cartridge was stored at − 20 °C until further processing. All the Sterivex cartridges were then randomly assigned to different extraction protocols after the filtration step to reduce bias on filtration order.

### DNA extraction and purification

All the connectors, stoppers, and silicon tubes were decontaminated by bleaching before use^5^. The RNAlater in the Sterivex cartridges was thawed on ice and removed by aspiration through the outlet. A silicon tube (1.5 cm; i.d. 3 mm; o.d. 9 mm) was used to connect a Luer-Lock adaptor (VRF306, AS ONE, Osaka, Japan) to the outlet such that both inlet and outlet can be sealed by Luer-Lock stoppers (VRMP6, AS ONE, Osaka, Japan). We tested three protocols that were modified from a published method that was widely used in the eDNA field^13^. The following reagents are based on the Qiagen DNeasy Blood and Tissue Kit (ThermoFisher Scientific, Waltham, MA, USA), with additional reagents (e.g. Buffer AL and proteinase K) purchased from the same sources when necessary. The major modifications are summarized in Fig. 7. For Protocol 1, we followed the original extraction protocol^13^. A lysis buffer mix (PBS 220 μL, Buffer AL 200 μL, Proteinase K 20 μL; 440 μL total volume) was introduced to the cartridge through the inlet. Both ends of the cartridge were sealed by Luer-Lock stoppers and the cartridge was incubated at 56 °C for 30 min with mild rotation. In Protocol 2, approximately four-times volume (2 mL) of lysis buffer mix (PBS 990 μL, Buffer AL 910 μL, Proteinase K 100 μL) was introduced through the inlet. After sealing the ends, the cartridge was incubated at 56 °C for 30 min without rotation. The rotation was omitted because the filter surface was completely covered by the lysis buffer mix. In Protocol 3, 2 mL lysis buffer mix (PBS 990 μL, Buffer AL 910 μL, Proteinase K 100 μL) was introduced into the cartridge through the outlet using a 2.5 mL syringe (Terumo Corporation, Tokyo, Japan) to flush the cartridge in a reverse direction. After sealing the ends, the cartridge was incubated at 56 °C for 30 min without rotation.

After the incubation, each Sterivex cartridge was placed in a spin column (maxi spin, flat bottom, Ciro Manufacturing Corporation, Deerfield Beach, FL, USA) attached to a 50 mL centrifuge tube, with the cartridge inlet facing downward. The unit was centrifuged with a swing-type rotor at 2500*g* for 10 min at 25 °C to elute the content. The cartridge and spin column were removed and molecular grade ethanol (99.5%, Fujifilm Wako Pure Chemical Corporation, Osaka, Japan) was added to one-third of the final volume (200 μL for Protocol 1 and 1 mL for Protocols 2 and 3). The mixture was loaded on a DNeasy Blood and Tissue Kit column attached to a vacuum manifold and the column was washed by 0.8 mL AW1 buffer and 0.8 mL AW2 buffer sequentially. The DNeasy column was dried by centrifugation at 17,700*g* for 2 min. Adsorbed DNA was eluted from the column with 75 μL AE buffer twice to maximize recovery. A total of 150 μL sample was collected from each extraction. Six replicates of Sterivex cartridges were used for each protocol. To test the extraction efficiency, each Sterivex cartridge was extracted 3 times and the eluted samples were analyzed separately.

An aliquot of DNA sample (100 μL) was further purified by Qiagen DNeasy PowerClean Pro Cleanup Kit (ThermoFisher Scientific, Waltham, MA, USA) according to the manufacturer’s protocol.

To test the binding capacity of the DNeasy column, we performed an additional experiment to determine whether the quantity of sample input will have a saturating effect on the column binding. We prepared a high sample input group by filtering 2 L of neat salmon seawater per Sterivex cartridge, while 1 L of 10×-diluted salmon seawater per cartridge was considered as a low sample input group. The extraction was performed using Protocol 3. Since we hypothesized that the binding capacity of one DNeasy column could be saturated by high input, we compared the eDNA extracted by a single column or double columns connected in series. In the case of double columns, the elution was performed individually for the upper and lower columns, and the eluates were analyzed separately.

### Chum salmon eDNA quantification

The eDNA was measured by quantitative PCR (qPCR) as described in a previous study^5^, with slight modification. Instead of using ABI TaqMan Environmental Master Mix 2.0 (Applied Biosystems, Foster City, USA), Takara Probe qPCR Mix (Takara Bio Incorporation, Kusatsu, Japan) was used as we showed that this reagent has a higher capacity to resist inhibition by environmental contaminants in the PCR reaction (see Results). We compared the resistance to contaminants by Environmental Master Mix and Takara Probe qPCR Mix according to the spiking/dilution methods described previously^5^, using the environmental samples collected from Murohama Bay of Otsuchi, which contained high levels of PCR inhibitors.

### Method validations in field samples

Thirty-four random seawater samples (1 L each) were collected from the Otsuchi River mouth (141′26.917 E, 39′55.021 N), Iwate Prefecture between March 15th–19th, 2020. Negative control was prepared by filtering distilled water (1 L). The water samples were filtered and treated with RNAlater as described in previous section. The cartridges were extracted according to the Protocol 3 described in previous section. To test the extraction and purification efficiencies, 10^10^ copies of a control plasmid (self-ligated pGEMT-easy, Promega, Madison, WI, USA) were added to each 2 mL lysis buffer mix (PBS 990 μL, Buffer AL 910 μL, Proteinase K 100 μL) at the beginning of the extraction. The extraction efficiency was calculated from ratio of control plasmid quantified from the DNeasy-extracted samples relative to input quantity. Real-time PCR for control plasmid was designed at the M13 regions: forward primer (pGEMT-F), 5′-TTTCCCAGTCACGACGTT-3′; reverse primer (pGEMT-R), 5′-TTCACACAGGAAACAGCTATGA-3′; probe (pGEMT-probe), 56/FAM/ACGCGTTGG/ZEN/ATGCATAGCTTGAGTA/3lABkFQ (Integrated DNA Technologies Inc., Coralville, IO, USA). Chum salmon eDNA concentrations of both DNeasy extracted samples and samples further purified by PowerClean Pro Cleanup Kit were measured to estimate the recovery rate of PowerClean purification. To estimate the PCR inhibitor effects from samples without purification by PowerClean Pro Cleanup Kit, an additional assay was performed with 10^5^ copies of chum salmon standard spiked into each PCR reaction. The chum salmon standard used in spiking is the same plasmid DNA^5^ used for constructing the standard curve. A reduction in spiked values calculated by subtracting the spiked copy number to original copy number indicates that PCR reaction was not optimal.

$$ {\text{Detection}}\,{\text{rate}}\,{\text{of}}\,{\text{spiked}}\,{\text{DNA}} = \frac{{\left( {{\text{copy}}\,{\text{in}}\,{\text{spiked}}\,{\text{sample}} - {\text{copy}}\,{\text{in}}\,{\text{original}}\,{\text{sample}}} \right)}}{{\left( {10^{5} \,{\text{copy}}\,{\text{of}}\,{\text{spiked}}\,{\text{DNA}}} \right)}} \times 100\% $$

Detection rates lower than 100% indicate that the PCR reaction could be affected by PCR inhibitors in environmental samples.

To test whether addition of bovine serum albumin (BSA) can further protect the real-time PCR reaction from non-specific inhibition, a final concentration of 1 μg/μL BSA (A4161, Sigma-Aldrich, St. Louis, MO, USA) was added to each Takara Probe qPCR Mix reaction. Real-time PCR was performed on the serially-diluted inhibitor-rich sample and the slope of the dilution curve was determined. PCR efficiencies were calculated according to the following equation (Efficiency = 10^−1/slope^)^27^. As we found improved quantification from BSA experiment, we further tested chemical additives including 2% DMSO (D2650-5X Sigma-Aldrich, St. Louis, MO, USA), 0.5% Tween 20 (P9416 Sigma-Aldrich, St. Louis, MO, USA), 0.01% Formamide (F9037 Sigma-Aldrich, St. Louis, MO, USA), and a protein-based additive GP-32 protein (Nippon Gene, Toyama, Japan) at 0.1 μg/μL final concentration^28^. A pooled eDNA sample was made from mixing some inhibitor-rich samples from the 34 random environmental samples, and subsequently used in this preliminary test. The PCR amplification plots were compared between chum salmon standard and the pooled sample. Slope of the PCR amplification plot was obtained by linear regression on the steepest linear portion of the curve. PCR reaction was compromised by the inhibitor when we observed a reduction in saturated fluorescence and a flatten slope of the linear portion of the amplification plot of the pooled sample in comparison to those of standard. We considered that the rescue from reduction in saturated fluorescence and slope change are indicating an increase in resistance to PCR inhibitors by the additive.

### Statistical analysis

The concentrations of salmon eDNA extracted by various protocols were analyzed by two-way ANOVA followed by Tukey’s multiple comparisons (GraphPad Prism Ver. 6 for Windows, San Diego, CA, USA). Statistical significance (*p* < 0.05) among various groups was denoted by alphabet letters. Purification efficiency of PowerClean Pro Cleanup Kit was calculated from paired samples quantified before and after the purification. Pearson’s correlation was performed to test whether the pre-purified quantity has a correlation to the purification efficiency in field water samples.
